# Protocol for detecting palmitoylation of high-molecular-weight rat synaptic proteins via acyl-PEG labeling

**DOI:** 10.1016/j.xpro.2024.103296

**Published:** 2024-09-06

**Authors:** Busra Perihan Yucel, Jeremy Martin Henley, Kevin Anthony Wilkinson

**Affiliations:** 1 Centre for Synaptic Plasticity, School of Biochemistry, Biomedical Sciences Building, University of Bristol, University Walk, BS8 1TD Bristol, UK; 2 Centre for Synaptic Plasticity, School of Physiology, Pharmacology and Neuroscience, Biomedical Sciences Building, University of Bristol, University Walk, BS8 1TD Bristol, UK

**Keywords:** Cell culture, Neuroscience, Protein expression and purification

## Abstract

Here, we present an optimized acyl-PEGyl exchange gel shift (APEGS) assay to monitor palmitoylation of high-molecular-weight proteins from primary neuronal cultures. We describe steps for culturing cortical neurons from rat embryos and expressing proteins of interest. We then detail procedures for employing a fatty acyl exchange technique wherein hydroxylamine is used to cleave palmitic acid from the palmitoyl-thioester bond, exposing cysteine residues that are subsequently labeled with methoxy polyethylene glycol maleimide (mPEG-MAL-10k).

For complete details on the use and execution of this protocol, please refer to Yucel et al.^1^

## Highlights

• Preparing primary rat cortical cell cultures
• Expressing tagged proteins with lentiviral vectors
• Detecting palmitoylation of high-MW synaptic proteins using a modified acyl-PEG assay
• Screening palmitoylated proteins via size separation on SDS-PAGE

## Abstract (editor highlights)

Publisher’s note: Undertaking any experimental protocol requires adherence to local institutional guidelines for laboratory safety and ethics.

## Graphical abstract

**Figure:**
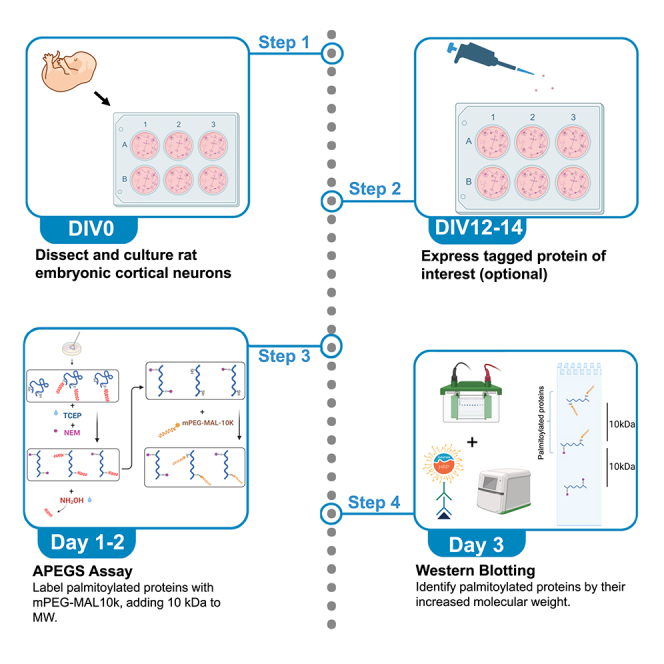

## Before you begin

Identifying palmitoylation, particularly of the proteins with low and/or transient levels of palmitoylation is challenging,^2^ and palmitoylation can be activity-dependent, thus requiring the experiment to be tailored to the individual target protein. Nonetheless, a range of detection assays have been developed, each of which has advantages and disadvantages.^3^

The approaches to detect palmitoylation vary, including metabolic labeling with radioactive palmitate ([3H], [14C], or [125I]),^4^ and using non-radioactive chemical probes via click-chemistry.^5^ These probes feature small chemical tags (alkyne or azide) at the fatty acid ends, minimizing interference with palmitoylation enzymes. Alternative techniques, such as the ‘acyl group exchange’ method, involve selective cleavage of the thioester bond between the acyl group and cysteine using neutral hydroxylamine. This yields free thiol groups, which can be captured using thiol-reactive biotin derivatives, thiol-sepharose beads, or PEG. These methods are referred to as ABE (Acyl-Biotin Exchange), Acyl-RAC (Acyl-Resin Assisted Capture), or APEGS (Acyl-PEGyl Exchange Gel Shift) assays, respectively.^6^^,^^7^^,^^8^^,^^9^^,^^10^^,^^11^

APEGS assays can be used with gel electrophoresis or mass spectrometry for quantification and identification of palmitoylated proteins, and can also determine palmitoylation stoichiometry, which is crucial for understanding its functional significance.^6^^,^^7^^,^^8^^,^^9^^,^^12^^,^^13^ However, it is important to note that labeling efficiency may vary in APEGS assays depending on protein size and structure, necessitating reaction condition optimization.^9^^,^^14^ Although the APEGS assay has been used to investigate the palmitoylation of synaptic proteins, including PSD95, AMPA, and NMDA receptors,^15^^,^^16^^,^^17^ previously published APEGS assays were unable to detect KAR subunit GluK2 palmitoylation.^9^^,^^11^

Here we describe an improved APEGS assay developed to investigate palmitoylation of the KAR subunit GluK2, which has a M_r_ >100 kD. We use GluK2 as a ‘proof of concept’ substrate to demonstrate the utility of the technique for probing possible palmitoylation of other high molecular weight proteins. In brief, we combined aspects of two previously reported protocols^9^^,^^11^ and tested a range of reagent concentrations and incubation times. Optimization of these parameters resulted in an assay that consistently and robustly allowed detection of GluK2 palmitoylation.^1^ This protocol details an optimized APEGS assay using primary neuron cultures, however it can also be applied to other cell types and tissues.

### Institutional permissions

All the animal experiments and procedures were performed in compliance with the UK Animal Scientific Procedures act (1986) and were guided by the Home Office Licensing Team at the University of Bristol. All animal procedures relating to this study were approved by the Animal Welfare and Ethics Review Board at the University of Bristol (approval number UIN/18/004).

## Key resources table

**Table:** 

REAGENT or RESOURCE	SOURCE	IDENTIFIER
**Antibodies**		

Anti-myc (Rb) (used at 1:2,000)	Cell Signaling Technology	71D10
Anti-GluR6/7 (b), clone NL9 (used at 1:5,000)	Merck Millipore	04-921

**Chemicals, peptides, and recombinant proteins**		

Poly-L-lysine solution	Merck	P4707
Boric acid, 98%	Merck	B0394
Sodium tetraborate (Borax)	Merck	221732
Triton X-100	Thermo Fisher Scientific	28313
Sodium dodecyl sulfate (SDS)	Merck	L3771
Bromophenol blue	Merck	B8026
2-Mercaptoethanol (βME)	Merck	97622
Tris-(2-Carboxyethyl) phosphine (TCEP) Bond-Breaker	Merck	77720
Hydroxylamine solution (NH_2_OH)	Merck	438227
Tetramethylethylenediamine (TEMED)	Merck	M3148-100ML
Methoxypolyethylene glycol maleimide (MPEG) – 10k	Merck	99126-64-4
N-ethylmaleimide (NEM)	Merck	04256-5G
Ethylenediaminetetraacetic acid (EDTA)	Merck	E5134-506
Chloroform, 99+%	Merck	372978
Methanol, 99.8%	Merck	34860
PageRuler prestained protein ladder, 10–180 kDa	Thermo Fisher Scientific	26616
Classico western HRP substrate, 500 mL	Immobilon	WBLUC0500
Crescendo western HRP substrate, 500 mL	Immobilon	WBLUR0500
Forte western HRP substrate, 500 mL	Immobilon	WBLUF0500
SuperSignal West Femto maximum sensitivity substrate	Thermo Fisher Scientific	34096
Hank’s balanced salt solution (HBSS)	Gibco	24020117
Neurobasal medium (NBA)	Gibco	21103049
Trypsin-EDTA	Gibco	25300096
GlutaMAX	Gibco	35050061
Horse serum	Gibco	26050088
B27 supplement	Gibco	17504044
Penicillin-Streptomycin (5,000 U/mL)	Gibco	15070063
Complete protease inhibitor cocktail	Merck	11697498001
Phosphate-buffered saline (PBS)	Gibco	14200-059
HyClone sterile purified water	Cutiva	SH3052902
Glycine	Severn Biotech	30-21-60
Stripping buffer	Thermo Fisher Scientific	46430
Ammonium persulfate (APS)	Thermo Fisher Scientific	17874
TRIS-Base	Merck	T1503-1KG
AcrylaGel	GeneFlow	ZT1267631S

**Deposited data**		

Western blots	Yucel et al.^1^	https://doi.org/10.3389/fnmol.2023.1270849

**Experimental models: Organisms/strains**		

E17 embryos of adult Han Wistar female rats	Charles River	Han Wistar

**Recombinant DNA**		

pXLG3-based lentiviral construct expressing a control, non-targeting shRNA (SCR; target sequence AATTCTCCGAACGTGTCAC) and GFP	Yucel et al.^1^	https://doi.org/10.3389/fnmol.2023.1270849
pXLG3-based lentiviral construct expressing an shRNA targeting GluK2 (target sequence GCCGTTTATGACACTTGGA) and shRNA-resistant YFP-Myc-tagged GluK2 (wild-type or non-palmitoylatable mutant)	Yucel et al.^1^	https://doi.org/10.3389/fnmol.2023.1270849

**Software and algorithms**		

Image Studio Lite	LI-COR	https://www.licor.com/bio/image-studio/

**Other**		

6-well cell culture plate	Greiner Cellstar	GD657160
Sonicator	Misonix Microson ultrasonic cell disruptor	NA
Hemocytometer	Improved Neubauer	NA

## Materials and equipment

**Table:** Plating media

Reagent	Final concentration	Amount
Neurobasal medium	N/A	455 mL
Horse serum	5%	25 mL
B27 supplement	2%	10 mL
Glutamax	1%	5 mL
Penicillin-streptomycin	1%	5 mL

Prepare under the hood, keep in fridge and use within 3 months.

**Table:** Feeding media

Reagent	Final concentration	Amount
Neurobasal medium	N/A	480 mL
B27 supplement	2%	10 mL
Glutamax	1%	5 mL
Penicillin-streptomycin	1%	5 mL

Prepare under the hood, keep in fridge and use within 3 months.

**Table:** Borate buffer

Reagent	Final concentration	Amount
Sodium tetraborate (Borax)	10 mM	1.0061 g
Boric acid	50 mM	1.5458 g
dH_2_O	N/A	Make up to 500 mL

Prepare and filter the solution under a laminar flow cabinet, store at RT for up to a year.

**Table:** Buffer A

Reagent	Stock concentration	Final concentration	Amount (μL)
SDS	10%	4%	4,000
EDTA	250 mM	5 mM	200
dH_2_O	N/A	N/A	Make up to 10,000

Can be kept at RT for months, add protease inhibitors if needed.

**Table:** Buffer B

Reagent	Stock concentration	Final concentration	Amount (μL)
NH_2_OH	16.5 M	2 M	242
EDTA	250 mM	5 mM	40
Triton	10%	0.2%	40
dH_2_O	N/A	N/A	Make up to 2,000

Prepare fresh every time. After adding NH_2_OH, adjust pH to 7.0 with HCl and add EDTA and triton.

**Table:** 1M NEM

Reagent	Final concentration	Amount
NEM	1 M	0.025 g
Ethanol	N/A	200 μL

Prepare fresh every time.

**Table:** Protease inhibitors (20x)

Reagent	Final concentration	Amount
Complete	20x	1 tablet
dH_2_O	N/A	2 mL

Can be stored in the fridge for 1–2 weeks.

**Table:** 50 mM MPEG-MAL 10K

Reagent	Final concentration	Amount
mPEG-MAL 10K	50 mM	250 mg
dH_2_O	N/A	1 mL

Vortex until it completely dissolves. Prepare a stock of 50 mM and make aliquots of 28 μL. Keep the aliquots at −80°C. Use one aliquot for 100 μL sample (final concentration 7 mM).

**Table:** 4**×** sample buffer

Reagent	Final concentration (mM)	Amount
TRIS pH 6.8	240	24 mL
SDS	8%	8 g
Glycerol	40%	40 mL
Bromophenol blue	0.009%	0.009 g
dH_2_O	N/A	Make up to 100 mL

Can be kept at RT for at least 1 year. Make 2x sample buffer when needed.

**Table:** 2× sample buffer

Reagent	Amount (μL)
4× sample buffer (SB)	500
β-mercaptoethanol	100
dH_2_O	400

4× SB can be kept at room temperature for the long term. 2× SB should be used within a week.

**Table:** 6% resolving gel

Reagent	Final concentration	Amount (mL) (15 mL–2 gels)
TRIS-HCl pH 8.8	375 mM	3.8
Acrylamide	6%	3
SDS	0.1%	0.15
APS	0.1%	0.15
TEMED	0.01%	0.012
dH_2_O	N/A	7.9

Prepare fresh every time.

**Table:** 5% stacking gel

Reagent	Final concentration	Amount (mL) (8 mL–2 gels)
TRIS-HCl pH 6.8	125 mM	1.3
Acrylamide	5%	1
SDS	0.1%	0.08
APS	0.1%	0.08
TEMED	0.01%	0.006
dH_2_O	N/A	5.5

Prepare fresh every time.

**Table:** SDS-PAGE running buffer

Reagent	Final concentration	Amount
TRIS	25 mM	30.25 g
Glycine	250 mM	187.65 g
SDS	0.1%	10 g
dH_2_O	N/A	Make up to 1,000 mL

Can be kept at RT for up to 1 month.

**Table:** SDS-PAGE Transfer Buffer

Reagent	Final concentration	Amount
TRIS	24 mM	29.04 g
Glycine	192 mM	144.1 g
Methanol	20%	200 mL
dH_2_O	N/A	Make up to 1,000 mL

Can be kept at RT for up to 1 month.

**Table:** 1.5M TRIS pH 8.8

Reagent	Amount
TRIS	363 g
dH_2_O	2,000 mL

Adjust pH to 8.8 with ∼44 mL HCl.

**Table:** 1 M TRIS pH 6.8

Reagent	Amount
TRIS	242 g
dH_2_O	2,000 mL

Adjust pH to 6.8 with ∼80 mL HCl.

**Table:** APS 10%

Reagent	Amount
Ammonium persulfate	5 g
dH_2_O	50 mL

Filter, make aliquots and store these at −20°C for up to 1 year.

## Step-by-step method details

### Primary cortical cell culture

**Timing: 2 days**

Primary neuronal cultures are a powerful and simple model system for studying neuronal biology. These cultures are obtained by dissecting the brains of mice or rats, typically from newborn pups or embryos. Different brain regions, such as the cerebellum, cortex, hippocampus, and peripheral components like the dorsal root ganglia, can be isolated and cultured. A comprehensive medium formulated for the optimal growth of brain-derived neurons *in vitro* is used. This sterile, liquid medium includes essential and non-essential amino acids, vitamins, organic and inorganic compounds, hormones, growth factors, and trace minerals.

1. Coat dishes with poly-L-lysine (PLL).
  a. To increase the adhesion of neurons to cell culture dishes, coat the wells of 6-well cell culture plates with 1 mL of 0.5 mg/mL PLL, diluted in borate buffer in a laminar airflow cabinet.
  b. Incubate the plates for 24 h in a 37°C/5% CO_2_ incubator.
  c. Wash the plates three times by adding 1 mL cell culture grade water to each well with a 10 mL pipette by using a Pipette boy, followed by aspiration.
  d. Add 1.5 mL plating media to each well.
  e. Leave the plates at 37°C/5% CO_2_ until plating the neurons.
2. Dissection of cortex from rat embryos.
  a. Sterilize the surgical tools and the bench with 70% ethanol solution before surgery.
  b. Anaesthetize a pregnant Han Wistar rat (at embryonic day E17) by using isoflurane and sacrifice humanely by cervical dislocation (Schedule 1 method).
  c. Sterilize the abdomen with ethanol (70%) and make a vertical cut through linea-alba to expose the abdominal cavity.
  d. Remove the uterus, containing the embryos, leaving both ovaries at the end of the uterus behind.
  e. Keep the embryos in Hank’s Buffered Saline Solution (HBSS) at RT throughout dissection.
  f. Remove the embryos from the uterine sacs and decapitate using scissors.
  g. Using sharp forceps under a dissecting microscope, remove the brains from the heads.
  h. Remove the meninges from both hemispheres, dissect cortex, and place into separate 35 mm dishes containing HBSS.
3. Culturing cortical neurons.
  a. Transfer dissected cortices to a 50 mL falcon tube, under a laminar airflow cabinet.
  b. With a sterile scalpel blade, gently score the surface of the cortices to increase the surface area to improve the access of trypsin.
  c. Wash the tissue three times by addition of HBSS (30 mL).
    ***Note:*** Allow pellet to yield by gravity.
  d. Aspirate the supernatant.
  e. Incubate cortices for 15 min in 30 mL HBSS containing 0.005% trypsin/EDTA, in a 37°C water bath.
    **CRITICAL:** Gently invert the tubes every ∼3 min to increase enzyme-substrate interaction.
  f. Wash cortices.
    i. Three times with HBSS (30 mL).
    ii. Once with plating media (5 mL).
    iii. Aspirate the media.
  g. Dissociate the cortices.
    i. Add plating media (5 mL),
    ii. Triturate with a 5 mL serological pipette on a Pipetboy.
      **CRITICAL:** Push the tip of the pipette against the bottom of the tube to force the cortices through the gap and perform around 14 up and down strokes to break up the tissues.
4. Determine cell concentration using a hemacytometer.
  a. Dilute the cortical cell suspension with a plating medium to a final volume of 20 mL.
  b. Gently pour the cortical suspension through a 70 μm cell strainer into a new sterile 50 mL Falcon tube.
  c. Count cortical cells with a hemocytometer and seed 500,000 cells into each well of a 6-well dish with 1.5 mL plating media (37°C) and incubate in a 37°C/5% CO_2_ incubator.

***Note:*** In this step you can check cell viability, cell number, and concentration.

5. Feed the cells.
  a. 2 h after seeding the cells, replace the plating medium with feeding media (2 mL) and place in a 37°C/5% CO_2_ incubator.
  b. After 7 days, feed the neurons by addition of a further 1 mL feeding media and return to the incubator until needed.
6. Viral transduction of neurons (optional).
  ***Note:*** For viral transduction of neurons, we followed a previously published lentivirus protocol.^18^
  a. The aliquots of lentiviruses were stored at −80°C and thawed before use in a 37°C water bath.
  b. Knockdown (KD)-rescue lentivirus-transduced cells expressing YFP-myc-GluK2, or the non-palmitoylatable point mutant YFP-myc-GluK2-C858A/C871A (GluK2-C2A), were left for 7 days prior to experiments in a 37°C/5% CO_2_ incubator.

### Acyl-PEG labeling assay

**Timing: 2–3 days**

**Figure 1:**
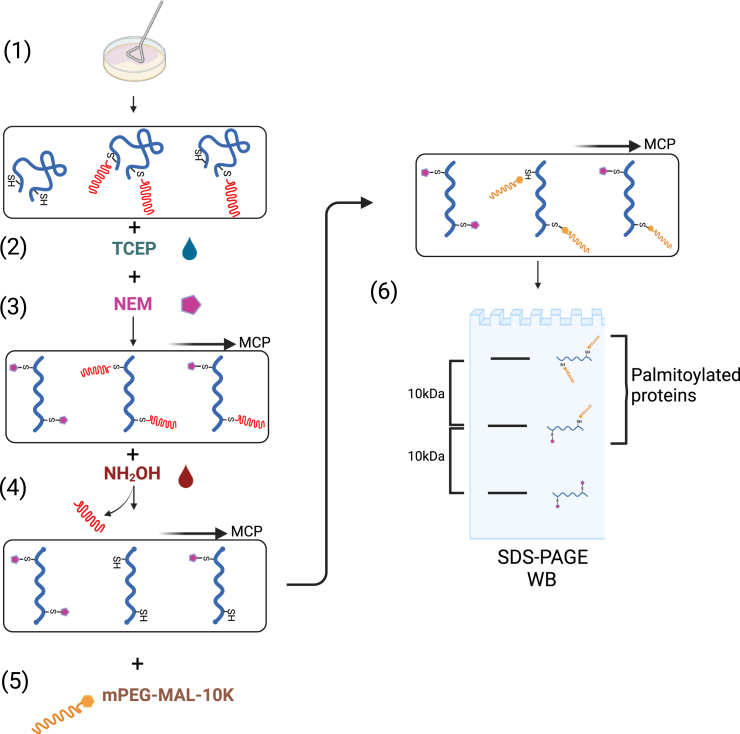
Steps of the APEGS assay Cells are lysed with Buffer A, followed by sonication and centrifugation (1). Lysates are incubated with 25 mM TCEP for 1 h to reduce disulfide bonds (2); lysates are then treated with 50 mM NEM overnight to block free cysteine residues (3); methanol-chloroform precipitation (MCP) is performed to remove NEM (3–4); palmitate moieties are then cleaved by addition of 2 M NH_2_OH (hydroxylamine) for 1 h (4); a second MCP is performed to remove hydroxylamine (4 and 5); formerly-palmitoylated cysteine residues are then labeled with 7 mM MPEG-MAL-10K for 2 h (5); a third MCP is performed to remove excess MPEG-MAL-10K. Samples are then run on a WB after boiling with sample buffer at 95°C for 3 min (6).

The Acyl-PEG labeling assay is an advanced technique used to monitor the palmitoylation status of proteins. This method involves the selective cleavage of the thioester bond between the palmitoyl group and the cysteine residue using neutral hydroxylamine. This reaction exposes free thiol groups on the protein, which are then labeled with a thiol-reactive polyethylene glycol (PEG) derivative, such as methoxy polyethylene glycol maleimide (mPEG-MAL-10k) (Figure 1). The addition of PEG increases the molecular weight of the protein by approximately 10 kDa, facilitating the detection of palmitoylation through a noticeable band shift during SDS-PAGE.

7. Preparation of the lysates.
  a. Lyse neurons in 100 μL Buffer A per well in 6-well dishes at RT.
    **CRITICAL:** Buffer A contains 4% SDS, which will freeze immediately on ice. Therefore, this step must be performed at RT, as should the rest of the steps in the APEGS assay.
    i. Scrape the cells using a plastic cell scraper and transfer the cell suspension to a 1.5 mL Eppendorf.
    ii. Sonicate the cell suspension with 3 sets of 4 pulses with a sonicator.
    iii. Spin down the samples for 20 min at 16000 *g* in a microcentrifuge at RT. Transfer the supernatant to a new 1.5 mL Eppendorf.
      **CRITICAL:** The volume of the lysate should be 100 μL to allow efficient methanol-chloroform precipitation by centrifuging samples in a 1.5 mL Eppendorf.
8. Measure the protein concentrations.
  a. Perform a BCA assay following the manufacturer’s protocol.
    i. Read the sample absorption using a plate reader (Versamax Microplate reader, Molecular Devices) at 562 nm wavelength.
    ii. Make a standard curve using 5 different concentrations (0, 0.125, 0.25, 0.5, 0.75, 1 mg/mL) of the BSA standard (2 mg/mL) solution provided with the BCA kit.
    iii. Calculate sample concentrations using the standard curve.
  b. Adjust the concentration of each sample to **2 mg/mL.**
9. Reduction of sample proteins and blocking of free cysteine residues.
  a. Add 5 μL 0.5 M TCEP (final concentration: 25 mM) to 100 μL of each sample (2 mg/mL) and incubate at 55°C in a water bath for 1 h.
  b. After incubating with TCEP, add 5.25 μL 1 M NEM (final concentration: 50 mM) to each sample to alkylate free cysteine residues and incubate overnight in a drawer at room temperature.
  c. Subject samples to the **1**^**st**^ **methanol-chloroform precipitation.**
    i. Add 400 μL methanol, 150 μL chloroform and 300 μL dH_2_O to each sample under a fume hood, close the lid, and shake the samples vigorously.
    ii. Centrifuge the samples for 5 min at 16000 *g* using a microcentrifuge.
    iii. Discard the upper layer carefully under a fume hood.
      **CRITICAL:** Be careful not to break up the pellet during this step.
    iv. Add 1 mL methanol and wash the pellet by carefully inverting the tube 2–3 times.
    v. Centrifuge the samples again at 16000 *g* for 5 min.
    vi. Discard the supernatant carefully.
    vii. Add 1 mL methanol again and repeat steps v. and vi.
      **CRITICAL:** Leave the tubes open under an air dryer for complete methanol evaporation (this step should optimally take 5 min). Drying the pellet will aid faster solubilization of the proteins in the next step.
      ***Optional:*** After any of the methanol-chloroform precipitation steps, you can freeze your pellets at −20°C and continue the rest of the assay within 24 h. Note, however, that in our hands freezing the sample decreases the efficiency of this protocol, but this likely depends on the specific protein under investigation and its palmitoylation state.
10. Cleavage of palmitic acid and labeling newly exposed cysteine residues.
  a. Add 25 μL Buffer A (without protease inhibitors) and incubate the samples in a water bath at 37°C for 10 min to solubilize protein pellets.
    **CRITICAL:** EDTA is crucial for effective thioester cleavage, but the protease inhibitor mixture should be excluded because these reagents can interfere with NH_2_OH reactivity in Buffer B.
    i. When pellets are completely solubilized, add 75 μL Buffer B to each sample to cleave palmitoylation thioester bonds.
    ii. Incubate samples in a water bath at 37°C for 1 h.
  b. Conduct the **2**^**nd**^ **methanol-chloroform precipitation** as described above in step 9.
    i. Solubilize the pellets in 100 μL Buffer A supplemented with 10 mM TCEP in a water bath at 37°C for 10 min.
11. Label newly exposed cysteines with mPEG-MAL.
  a. Take mPEG-MAL aliquots (one for each sample) from the −80°C freezer and thaw at 25°C.
    i. Add 28 μL of 25 mM mPEG-MAL (cut the top of the pipette tip) to sample solubilized in 100 μL Buffer A to label newly exposed cysteinyl thiols (final concentration 7 mM).
    ii. Leave the samples on a bench top shaker for 2 h at 25°C.
  b. Conduct the **3**^**rd**^ **methanol-chloroform precipitation** as described above.

***Optional:*** When pellets are completely dry, you can keep the samples in a freezer (−20°C) and perform sodium dodecyl sulfate–polyacrylamide gel electrophoresis (SDS-PAGE) and Western blotting (WB) later.

**CRITICAL:** The best way to preserve labeled cysteines is to keep samples as pellets until continuing to the SDS-PAGE and WB stages. Otherwise, due to the labile bond between the mPEG-MAL and the cysteine residues, some mPEG might be cleaved, and the result might not be indicative of the palmitoylation state of the protein of interest.

12. Sample preparation.
  ***Note:*** Ideally, samples should be run on a 6% gel. Pellets can be kept frozen at −20°C and solubilized a couple of days later (however, we do not recommend that they are left in the freezer for more than 2–3 days).
  a. Solubilize pellets with 60 μL 2x sample buffer without βME in a water bath at 37°C for 10 min.
    **CRITICAL:** It is important not to use βME initially because it might interfere with the BCA assay.
  b. Repeat the BCA assay to standardize the protein concentrations across all samples, aiming for all samples to have the same concentration (typically in the range **0.5–1 mg/mL**).
  c. Add 2% βME to each sample and boil the samples at 95°C for 3 min.
    ***Optional:*** If proteins are sensitive to high temperature boiling is not necessary.

### Monitoring protein palmitoylation using SDS-PAGE and western blotting (WB)

**Timing: 8 h**

In this method, proteins are first separated by size through SDS-PAGE, which ensures uniform negative charge and linearization of the proteins, allowing for effective separation based on protein molecular weight. After electrophoresis, proteins are transferred onto a membrane in a WB process. The membrane is then probed with specific antibodies that recognize the protein of interest.

13. Gel preparation and electrophoresis.
  a. Prepare a 6% acrylamide resolving gel between two 1.5 mm gel plates.
    i. Once set, wash 3 times with double distilled water and pour a 5% stacking gel.
    ii. Place into the gel a 1.5 mm 10 well comb.
    iii. Assemble the gels into an electrode after polymerization and insert the assembly into an appropriate electrophoresis tank.
  b. Fill the reservoirs with SDS-PAGE running buffer.
  c. Load the first well with 6 μL PageRuler Prestained protein ladder using a pipette tip and the rest of the wells with 20 μL sample.
  d. Run the electrophoresis at constant 100V.
    ***Optional:*** Once the samples have entered the resolving gel this can be increased to 150 V.
  e. Run the samples until the dye front reaches the bottom of the gel.
    **CRITICAL:** If the protein of interest has a molecular weight (M_r_) greater than 150 kDa, run the gels until the 70 kD protein ladder band reaches the bottom of the gel. This will allow sufficient separation of the non-palmitoylated and palmitoylated proteins.
  f. Stop the electrophoresis and continue with the protein transfer.
14. Transferring proteins to the membrane.
  a. Prepare the transfer materials.
    i. Activate freshly cut PVDF membrane in 100% methanol for a couple of seconds and then incubate it in SDS-page transfer buffer for 10 min at 4°C.
    ii. Soak filter paper and sponges in ice-cold transfer buffer.
  b. Separate the glass plates.
  c. Cut the stacking gel from the resolving gel and discard it.
  d. Assemble a transfer cassette with the resolving gel and PVDF membrane between filter paper and sponges.
    **CRITICAL:** Make sure transfer buffer contains methanol otherwise transfer will not be successful.
    **CRITICAL:** Prepare the transfer sandwich carefully following the order of cassette/sponge/filter paper/gel/membrane/filter paper/sponge/cassette, and ensure any bubbles are removed by gentle rolling. Cassettes should be clamped firmly, but not too tightly as this can damage the gel. If it is too loose and/or there are air bubbles, proteins will not transfer efficiently.
  e. Place the cassette in a transfer tank with the gel facing the cathode and the membrane facing the anode.
    i. Fill the tank with the transfer buffer.
    ii. Add an ice pack for keeping it cool.
    iii. Carry out the wet transfer at a constant 400 mA for 120 min with constant stirring.
      ***Note:*** A 120-min transfer allows enough time to transfer mPEG-conjugated proteins, as their molecular weight will be around 160–180 kDa in the case of GluK2. Less than 120 min might not result in sufficient transfer.
15. Western Blotting (WB) / Immunoblotting.
  a. Remove the PVDF membrane from the sandwich and place it in a WB box.
  b. Block the PVDF membrane in 6% low fat skimmed milk (in PBS-T (PBS + 0.001% Tween-20) for 1 h at RT.
  c. Incubate the PVDF membrane 1 h with primary antibody diluted to the appropriate concentration in 6% milk in PBS-T.
  d. Wash the membrane briefly with PBS-T (3 times) to remove excess antibody.
  e. Incubate with HRP-conjugated secondary antibody diluted 1:10000 in 6% milk in PBS-T for an hour.
  f. Wash the membrane with PBS-T (3 times for 5 min) on a rocker.
16. Chemiluminescence detection.
  a. To visualize protein bands, incubate the membrane with a chemiluminescence substrate for 1–2 min at RT.
    ***Note:*** We use a variety of substrates of differing sensitivity depending on the particular requirements of the experiment. From lowest to highest sensitivity, these are: Luminata Classico, Crescendo, Forte and Supersignal Femto.
  b. After removing the excess substrate, place the membrane between two pieces of clear plastic sheet.
    ***Optional:*** Signal can be detected using a Western blot imager, such as a LI-COR Odyssey or using X-ray film.
    ***Note:*** If weak or no signal is detected, the membrane can be washed briefly in PBS-T and incubated with a higher sensitivity chemiluminescence substrate.
  c. Record the signal by exposing the membrane in the chemiluminescence channel for 5 min, and the protein ladder can be detected using the 700-fluorophore channel for 0.5–1 min.
17. Stripping and reprobing of the membrane.
  a. After recording the results, wash the membrane with PBS-T (3 times) and incubate it with 5 mL Restore Stripping buffer for 20 min at 65 °C.
  b. Discard the stripping buffer, and wash the membrane 5 times (quick-washes) with PBS-T and leave it on a rocker for 10 min in PBS-T.
  c. Discard the PBS-T, add new primary antibody solution.
  d. Repeat rest of the WB steps to probe for any other proteins of interest.

***Note:*** Western blots can be stripped and reprobed several times to allow detection of multiple proteins of interest on the same membrane.

## Expected outcomes

**Figure 2:**
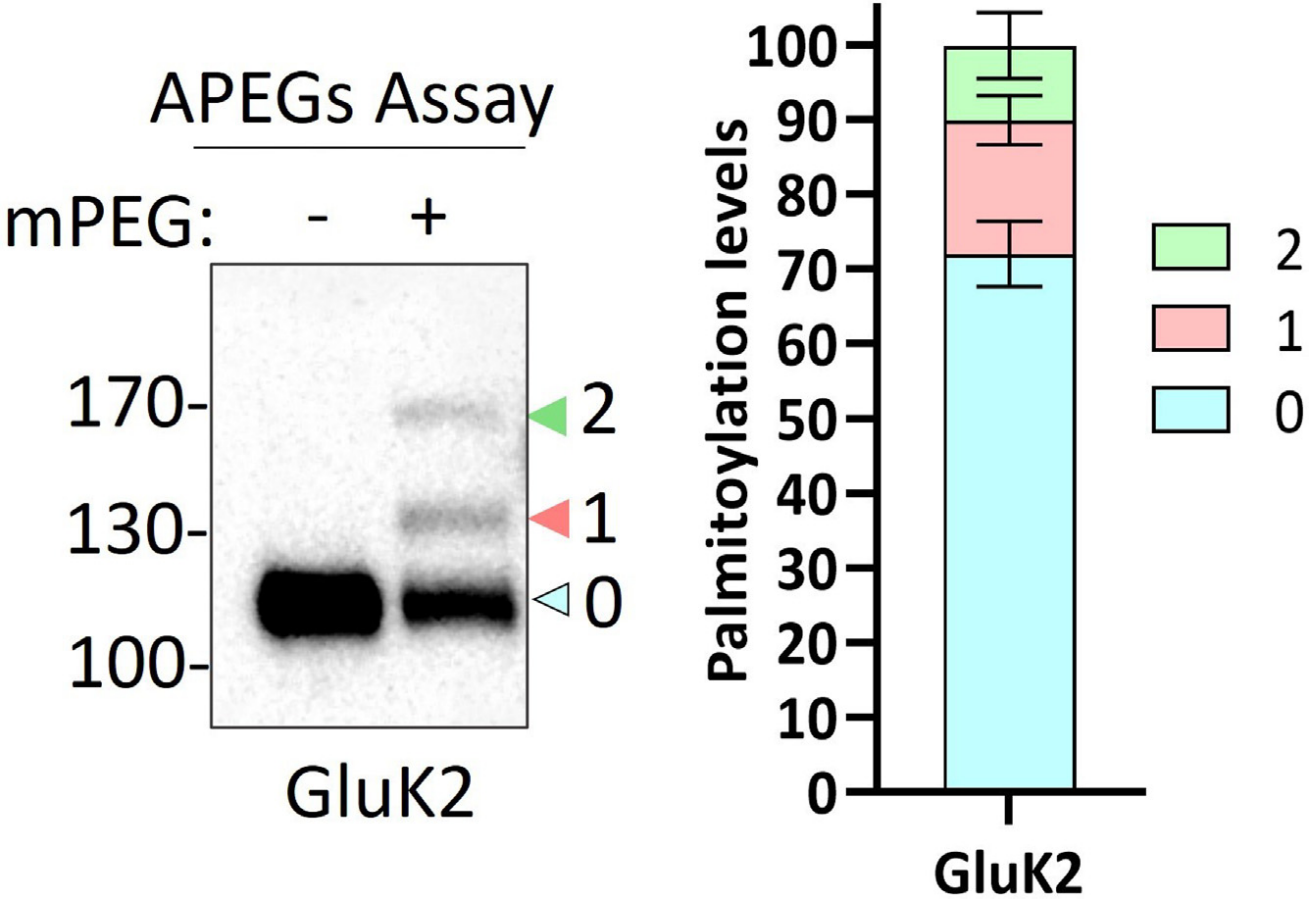
The palmitoylation of endogenous GluK2 in rat cortical neurons Rat cortical neurons were plated and subjected to the APEGS assay at DIV 19–21. Approximately 28% of the total GluK2 was palmitoylated at either one (∼18%) or two (∼10%) cysteines. In the analysis, “0” denotes non-palmitoylated GluK2, “1” denotes palmitoylation at one cysteine, and “2” denotes palmitoylation at two cysteines. *N* = 3 different dissections. Error bars = ±SEM. This figure is reproduced from Yucel et al.^1^

**Figure 3:**
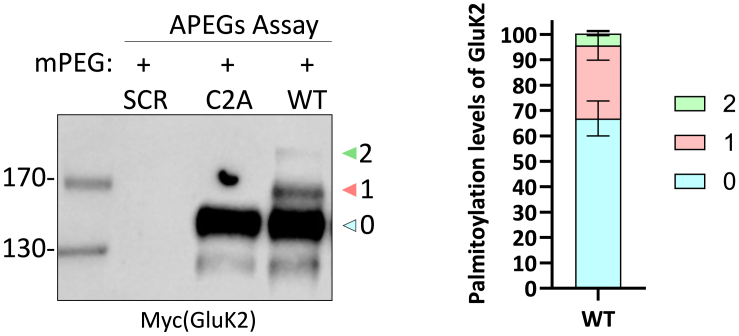
GluK2 is palmitoylated at both C858 and C871 residues Cortical neurons were incubated with lentiviruses expressing both a control shRNA and GFP (SCR) or an shRNA targeting endogenous GluK2, along with shRNA-insensitive YFP-Myc-tagged GluK2 wild type (WT) or a double cysteine mutant (C858A/C871A; C2A). Viruses were added at DIV 14, and cells were lysed 7 days later. Lysates were then subjected to the APEGS assay followed by Western blotting (WB). The GluK2 C858A/C871A mutant is not palmitoylated in neurons. Approximately 34% of GluK2 WT is palmitoylated, with ∼29% occurring at a single cysteine and ∼5% at both. “0” denotes non-palmitoylated GluK2, “1” denotes palmitoylation at one cysteine, and “2” denotes palmitoylation at two cysteines. *N* = 3 dissections. Error bars = ± SEM. This figure is reproduced from Yucel et al.^1^

This protocol allows detection and quantification of levels of palmitoylation of relatively high molecular weight (>100 kDa) proteins. Moreover, a 10 kDa band shift demonstrates single cysteine palmitoylation whereas a 20 kDa band shift signifies two cysteine palmitoylation events occurring simultaneously. Using this method, we demonstrated that GluK2 is endogenously palmitoylated (Figure 2) at two different cysteine residues (C858 and C871) (Figure 3), consistent with previous data obtained using radiolabeled palmitate.^1^^,^^4^

## Quantification and statistical analysis

The data acquired using a LI-COR Odyssey should be quantified using Image Studio Lite Version 5.2 as pixel signal intensity for each protein band. The signal intensity for the selected palmitoylated band should be calculated by subtracting the product of background and area from the main band. The values obtained can be copied to Microsoft Excel and the bands should be normalized to their native (non-palmitoylated) bands.

## Limitations

Potential limitations of this protocol include the requirement for multiple methanol-chloroform precipitation steps which, if not performed very carefully, can be a major source of error due to incomplete precipitation. In addition, the assay is unavoidably time-consuming. Finally, although the template detailed above represents a convenient starting point, it is ultimately necessary to validate and optimize the APEGS assay for each protein under investigation.

## Troubleshooting

### Problem 1

False positives on the blot.

### Potential solution

• Inefficient concentrations of NEM or a short incubation time could lead to inefficient blockage of cysteine residues, causing false positives. Having a non-palmitoylatable mutant and negative control (e.g., without mPEG or without hydroxylamine) will help to identify the correct bands. NEM concentration and incubation should be followed as mentioned above (See 9b at p10).

### Problem 2

Lack of higher M_r_ band(s) corresponding to palmitoylated substrate protein.

### Potential solution

• Do not add protease inhibitors to Buffer A. Protease inhibitors decrease the efficiency of hydroxylamine (See 9b).

### Problem 3

Higher M_r_ band(s) corresponding to palmitoylated protein of interest are faint and hard to detect.

### Potential solutions

• This may be due to loss of some palmitoylated proteins during the methanol-chloroform precipitation (MCP) steps. MCP needs to be performed carefully, and methanol washes are important to remove the NEM and hydroxylamine. While performing MCP try not to touch the protein pellet as much as possible (See 9c).
• Using heterologous systems with high overexpression makes it hard to see the comparatively low abundance palmitoylated bands. Try to control the protein expression levels by using knockdown-rescue methods or perform experiments at endogenous levels (See 6b).
• Ideally, samples should be run on a 6% gel immediately after the APEGS assay. However, pellets can be stored at −20°C and solubilized a couple of days later with sample buffer, as described in the protocol (we recommend they are not left in the freezer for more than 2–3 days) (See 12).
• To enhance blotting sensitivity, it can be helpful to begin with a larger amount of material, to increase the amount of protein loaded in the well, elevate antibody concentrations, use a more sensitive ECL substrate, and visualize bands with X-ray film.

### Problem 4

It is not possible to heat the protein of interest to 95°C for SDS-PAGE.

### Potential solution

• If your protein of interest cannot be heated in SDS-PAGE sample buffer due to aggregation, then you may not need to include this step. As an alternative, after adding the βME (sample preparation step 4), incubate the samples at RT overnight rather than heating to 95°C and run on an SDS-PAGE the next morning (See 12).

### Problem 5

Protein pellets do not dissolve after adding sample buffer.

### Potential solution

• Make sure to wash the pellet with methanol and dry the protein pellets after MCP under an air dryer or fume hood for 5 min to remove all the methanol (See 9c). Otherwise, it is hard to solubilize the pellets.

## Resource availability

### Lead contact

Further information and requests for resources and reagents should be directed to and will be fulfilled by the lead contact, Kevin Anthony Wilkinson (kevin.wilkinson@bristol.ac.uk).

### Technical contact

For any technical queries related to the protocol, such as clarification on methods, please contact Busra Perihan Yucel (busra.yucel@bristol.ac.uk).

### Materials availability

This study did not generate new unique reagents.

### Data and code availability

This study did not generate any unique datasets or codes.

## Acknowledgments

B.P.Y. was a PhD student funded by the Turkish Ministry of National Education. We are grateful to the BBSRC (BB/R00787X/1), MRC (MR/L003791/1), Leverhulme Trust (RPG-2019-191), and Wellcome Trust (105384/Z/14/A) for financial support. The graphical abstract and figures in this article were created using BioRender.com.

## Author contributions

B.P.Y. modified the APEGS assay and performed the experiments. B.P.Y. and K.A.W. contributed to designing and planning. K.A.W. provided the lentiviral constructs. All writers contributed to writing and/or editing the manuscript. J.M.H. and K.A.W. contributed to overall supervision.

## Declaration of interests

The authors declare no competing interests.
